# The burden of suicide across different altitudes: 11-year geodemographic analysis conducted in 221 cantons in Ecuador ranging from 0 to 4300 m of elevation

**DOI:** 10.1192/bjo.2024.736

**Published:** 2024-09-24

**Authors:** Esteban Ortiz-Prado, Juan S. Izquierdo-Condoy, Raul Fernandez-Naranjo, Jorge Vásconez-González, Sebastián Encalada, Johanna Mosquera, Simone Cordovez, Nicole Camino, Daniela Montenegro-Salazar, Ginés Viscor, Ana María Diaz, Clara Paz

**Affiliations:** One Health Research Group, Faculty of Medicine, Universidad de las Américas, Ecuador; Physiology Section, Department of Cell Biology, Physiology and Immunology, Universidad de Barcelona, Spain; Wellbeing, Health and Society Research Group, School of Psychology and Education, Universidad de Las Américas, Ecuador

**Keywords:** Suicide, high altitude, depression, public health, Ecuador

## Abstract

**Background:**

The World Health Organization and the Global Burden of Disease study estimate that almost 800 000 people die from suicide yearly. The role of non-traditional risk factors such as climate and high-altitude exposure are poorly understood.

**Aims:**

This study aims to determine a potential relationship between altitude exposure and suicide rates among 221 cantons located at different altitudes ranging from 0 to 4300 m.

**Method:**

We conducted an 11-year, country-wide, population-based analysis on age- and gender-standardised suicide rates in Ecuador, based on the official data from the National Institute of Statistics, using all available self-harm death codes (ICD-10 codes X60–X84).

**Results:**

A total of 11 280 cases of suicide were reported during 2011–2021. Suicide rates were higher among men (11.48/100 000). In terms of elevation, suicide rates were significantly higher among people from high-altitude cantons (3.7/100 000) versus those from low-altitude cantons. When applying the International Society Mountain Medicine categorisation, suicide rates were significantly higher at moderate- (4.3/100 000), high- (3.6/100 000) and very-high-altitude cantons (4.4/100 000) when compared with low-altitude locations (2.5/100 000).

**Conclusions:**

Ecuador is one of the few countries that has a vast range of cantons located at different altitudes. We found that living at higher elevations is positively associated with greater suicide rates. Although the rates are significantly greater as elevation increases, a clear linear relationship is not apparent, likely because of the interplay of socioeconomic factors, including urbanicity. The effect of chronic hypobaric hypoxia on mood cannot be ruled out, although the existence of causal mechanisms remains to be elucidated.

The World Health Organization (WHO) and the Global Burden of Disease (GBD) study estimate that more than 700 000 people die from self-inflicted injuries every year, translating to at least one death occurring every 40 s.^1^ This public health problem is one of the leading causes of death among men and women worldwide. For example, in the Americas, suicide rates among young people aged 20–24 years represent the third leading cause of death.^2^

Suicide is one of the most relevant global health problems, not only because of the burden of disease that has been increasing in recent decades, costing thousands of lives globally, but also because millions of years of life lost prematurely (YLL) are lost daily at a global level.^1^ In some countries, suicide is the third most common cause of mortality for younger populations, especially those aged 15–24 years.^3^ In the past 20 years, the suicide rate per 100 000 male children (10–14 years) has slightly decreased, whereas the rate for same-age girls has slightly increased, from 0.85 to 0.94 suicide per every 100 000 girls.^4^

Suicide is a multifactorial condition that is influenced by environmental, cultural, psychological and hereditary variables.^5^ Risk factors include being male (as global suicide rates have been estimated to be 18/100 000 for men and 11/100 000 for women),^6^ meeting diagnostic criteria for a psychiatric illness, having limited income, possessing guns, being incarcerated, unemployment and drug misuse.^5,7–11^

In terms of social risk factors, the lack of social capital, poverty, community violence, sexual abuse, trauma and discrimination^11^ are some of the well-known studied factors for suicide, whereas non-traditional risk factors have also been investigated.^12^ For instance, it has been suggested in recent decades that suicide rates may increase, at least partly, during winter, when exposed to harsh weather, constantly living in colder areas of the planet and more recently, among high-altitude dwellers.^13^

Recent studies have found a possible association between high suicide rates and living at high altitude.^14–16^ The mechanism underlying this relationship is not fully understood, however, it can be speculated that it depends on molecular factors triggered by hypobaric hypoxia or on social, cultural or political factors associated with high-altitude locations, such as social isolation, reduced access to healthcare and harsh weather pushing people to live in smaller social groups. Hypobaric hypoxia is a condition that commonly occurs at high altitudes, where the atmospheric pressure is lower than at sea level. This form of hypoxia can significantly impact neurophysiological functions, particularly altering levels of mood-regulating neurotransmitters like serotonin. Such alterations have been associated with depression and, consequently, suicide.^17,18^

When looking for biological plausibility and answers, some researchers have suggested that metabolic stress induced by mild hypoxia in those with mood disorders may be linked to suicide.^19^ It has been hypothesised that living in at high altitude may have an effect on the serotonin metabolism, reducing the synthesis of the 5-hydroxytryptophan (5-HTP), thus decreasing serotonin levels within the central nervous system.^20^ The study by Kious et al might direct our understanding in how low serotonin production might be linked to mood symptoms that are common among people residing at high altitude.^18^ It has also been reported that brain chemistry seems to be altered because of long-term exposure to higher altitude.^21^ DelMastro et al also reported significant correlations between high altitude and depressive and anxious symptoms, as well as sleep problems.^22^ In a recent study, Ortiz-Prado et al found that high-altitude natives have more unfavourable self-reported health states, lower scores in the 36-item Short Form Survey (SF-36) and tend to be more pessimistic than their low-altitude counterparts.^20^ Additionally, other authors point out that although hypoxia's effects on mood and sadness have been theorised as contributing elements, it is likely that a significant proportion of other individual and social factors play a larger role.^23^

Recently, a literature review on suicide and altitude found that elevation and other covariates are probably associated with a higher suicide rate. This review included 19 studies, from which 17 reported evidence of a positive correlation between altitude and increased suicide even with variations among studies.^24^ In some countries, it has been described that altitude could be associated with greater likelihood of suicide. For instance, Brenner et al reported a significant increase in suicides among high-altitude populations in the USA.^25^ In Ecuador, for example, an ecological study found that highland provinces reported greater suicide rates of suicide than those with lower altitudes (9 per 100 000 *v.* 4 per 100 000).^3^

Although most evidence suggests that high altitude is associated with a higher risk of suicide, very few countries have altitude variability cantons ranging from 0 to 4300 m to explore this relationship, as Ecuador does.

## Aims

We aim to study official national data on suicide rates and their characteristics across 221 cantons located at low, moderate, high and very-high altitudes.

## Method

### Study design

This is an ecological, nationwide analysis of the geographical distribution of suicide across 221 cantons from Ecuador, using information from the most recent years of available data (2011–2021).

### Population

Ecuador is in South America and, according to national census data, the total population surpassed 17.8 million people in 2021, with 51% being women and 49% being men. In terms of ethnicity, the majority of people are Mestizo (79.3%), followed by Afro-Ecuadorians (7.2%), Indigenous (7.1%), White or Caucasian descendants (6.1%) and other groups (0.4%). In terms of elevation, 60% of the population resides at low altitude (<1500 m), 10% at moderate altitude (1500–2500 m), 27% at high altitude (2500–3500 m) and 3% at very-high altitude (3500–5500 m) (see Supplementary File 1 available at https://doi.org/10.1192/bjo.2024.736).^26^

### Sample and setting

A country-wide comparison of the total number of intentional self-harm or suicide-related deaths from the 24 provinces and 221 cantons in Ecuador was performed, covering the period from 2011 to 2021. All suicide-related deaths were retrieved from the National Institute of Census and Statistics of Ecuador (INEC) annual mortality database.^27^ It is crucial to acknowledge that there might be cases that were not captured in the database for various reasons, such as underreporting or misclassification. Ecuador's political division comprises ten provinces in the highlands, seven on the coast, six in the Amazon region and one in the insular region of Galapagos. Every province has several political divisions called cantons, which are comparable to cantons elsewhere. The country has 141 low-altitude cantons, 28 moderate-altitude cantons, 41 high-altitude cantons and 11 cantons located at very-high altitude.

### Exposure

The association between altitude living and suicide mortality rate was analysed. The classification of low altitude (<2500 m) and high altitude (>2500 m) was used as a cut-off point for elevation exposure, whereas the classification offered by the International Society of Mountain Medicine (ISMM) for low altitude (<1500 m), moderate altitude (1500–2500 m), high altitude (2500–3500 m) and very-high altitude (3500–5500 m) was used to assess prevalence odds ratios at the different elevation zones.^26^

### Outcome

Suicide age-, gender- and altitude-adjusted mortality rates were calculated by using the total number of suicide-related deaths in Ecuador.

### Data source and description

All cases of suicide were classified according to the ICD-10. ICD-10 codes reflecting intentional self-harm (X60–X84) were identified through autopsy reports provided by the INEC as death certificates.^27^ Data on demographic variables such as gender, province elevation, educational attainment and marital status for all cases of suicide were included. The overall crude and age- and gender-adjusted mortality rates were calculated by using the National Demographic Projections to obtain the number of people at risk (Supplementary Table 1).

### Bias control

The results presented in this study are derived from the data officially registered on the government's official INEC website. To reduce the possibility of incurring in some degree of selection bias and because of the nature of the data, two researchers (E.O.-P. and R.F.-N.) independently downloaded the data-set and conducted the analyses. No individual manipulation was conducted, and the results were compared to ensure complete agreement.

### Data analysis

The variables analysed in this study included place of residence of the deceased (canton, province and region), age at the time of death, gender, educational attainment, marital status, mechanism of suicide and ethnic group. The suicide rate was gender- and age-standardised using projection data by canton and province according to the 2010 census. Mortality (suicide) rates were computed by age, gender, geographic location and their corresponding population. All cases were classified across 17 age groups. For association, *t*-tests were used to assess differences between suicide rates and descriptive variables.

To ensure the robustness of our findings in the context of multiple comparisons, the Bonferroni correction was applied to the *P*-values obtained from these *t*-tests. This correction is particularly crucial, given the comprehensive nature of our comparisons across different demographics and geographic categories. The Bonferroni method adjusts the significance threshold to account for the number of comparisons, thereby reducing the likelihood of type I errors (false positives).

The data analysis was performed with the SPSS statistical package software for Macintosh (version 29) and ‘R’ version 3.6.2 (R Foundation for Statistical Computing, Vienna, Austria; https://cran.r-project.org). Figures and graphs were plotted with Prism 8 GraphPad Software version 8.2.0 (GraphPad Software, San Diego, USA; https://www.graphpad.com). Basic cartography maps were generated with QGIS Development Team 2.8 (QGIS Development Team; https://qgis.org). The specific details of the Bonferroni correction, including the adjusted significance level, are elaborated in the Results section.

### Ethical considerations

The study was conducted using anonymised and publicly available data. In accordance with the guidelines of the Ministry of Public Health of Ecuador, ethical approval is not required for such studies. To provide further transparency, we applied to the Ethics Committee for Research on Human Subjects of the Universidad de Las Américas (CEISH-UDLA) and received a written exemption (reference 2023-EXC-008) confirming that the project is exempt from CEISH evaluation as per current legal regulations.

## Results

### General description

#### Age and gender

Based on our analysis, there were a total of 11 280 registered suicide deaths (ICD-10 codes X60–X84) in Ecuador between 2011 and 2021. Among these, 8682 suicides occurred in men, representing 77% of the total, and 2598 suicides were reported in women, making up the remaining 23%.

The overall gender-adjusted mortality rate was 9.6 per 100 000 for men and 2.8 per 100 000 for women. Notably, the male suicide rate was four times higher than that of females, and this difference was statistically significant (*P* < 0.001).

**Fig. 1:**
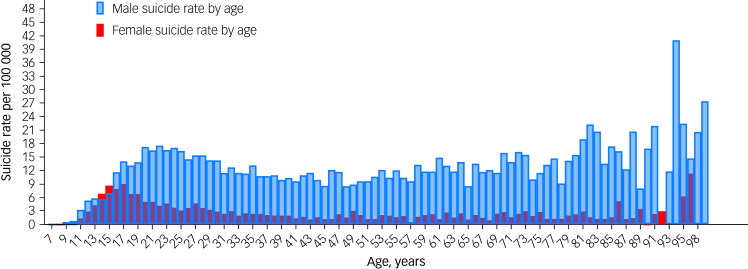
Suicide rate by age frequency from 2011 to 2021.

The average age was 36.5 years (s.d. = 21.31) for men and 29.57 years (s.d. = 17.13) for women. Regarding the number of suicides by age, most suicides occurred between 15 and 39 years of age, representing 59% for men (*n* = 5142) and 66% for women (*n* = 1707). At 14–15 years of age, women had higher suicide rates than men (7.9/100 000 *v.* 6.3/100 000), with this difference being statistically significant (*P* < 0.001) (Fig. 1).

### Geodemographic distribution

#### Trends by province and canton

Ecuador's provinces and cantons with the highest adjusted suicide rates per 100 000 inhabitants, as per the individual's recorded place of residence, revealed significant variations. In terms of provinces, Azuay, Cañar and Orellana stood out, with rates of 9.78/100 000, 9.5/100 000 and 9.32/100 000, respectively. Delving into the canton-level analysis, Chilla, Isabela and Quilanga exhibited the highest suicide rates, with rates of 867.96/100 000, 542.09/100 000 and 484.83/100 000, respectively (Table 1 and Fig. 3).

**Table 1:** Total number of cases, incidence rates/100 000 and suicide rates for Ecuador provinces from 2011 to 2021

Province	Cases	Crude rate/ 100 000	Adjusted rate/ 100 000
Azuay	1006	11.03 [10.07; 12.4]	9.78 [8.69; 11.03]
Bolivar	226	10.16 [10.09; 10.7]	9.5 [9.26; 10.23]
Cañar	260	9.02 [7.35; 11.03]	8.01 [6.64; 9.78]
Carchi	178	8.84 [6.54; 10.23]	8.16 [5.89; 9.55]
Chimborazo	437	7.85 [7.25; 8.9]	7.24 [6.39; 8.34]
Cotopaxi	474	9.29 [7.14; 11.14]	8.38 [6.51; 10.36]
El Oro	515	6.83 [5.7; 7.78]	6.09 [5.39; 6.7]
Esmeraldas	237	3.55 [2.76; 4.16]	3.16 [2.29; 3.84]
Galapagos	18	5.36 [3.2; 7.24]	4.51 [2.71; 6.62]
Guayas	1801	3.96 [3.61; 4.63]	3.53 [3.23; 3.99]
Imbabura	357	7.23 [6.12; 9.1]	6.52 [5.2; 8.4]
Loja	361	6.51 [5.77; 7.83]	5.91 [5.48; 6.81]
Los Rios	622	6.45 [5.76; 7.36]	5.78 [5.07; 6.53]
Manabí	804	4.85 [4.12; 5.9]	4.43 [3.67; 5.27]
Morona Santiago	143	7.24 [5.43; 9.62]	5.96 [4.57; 8.26]
Napo	145	10.77 [7.03; 13.43]	9.26 [6.04; 11.47]
Orellana	173	10.32 [9.07; 12.6]	9.32 [7.86; 11.43]
Pastaza	77	6.92 [4.62; 10.27]	5.79 [3.62; 8.61]
Pichincha	2178	6.53 [6.4; 8.11]	5.71 [5.31; 6.99]
Santa Elena	112	2.76 [2.34; 3.27]	2.35 [1.89; 2.77]
Santo Domingo	339	7.19 [7.17; 8.44]	6.14 [5.84; 7.5]
Sucumbíos	215	9.35 [7.94; 9.85]	7.96 [6.15; 8.43]
Tungurahua	508	8.2 [6.97; 10.25]	7.6 [6.63; 9.44]
Zamora Chinchipe	90	7.31 [3.98; 9.33]	6.16 [3.33; 7.88]
Missing data	4		
Total	11 280		

In brackets are indicated rates by gender [men; women].

**Fig. 2:**
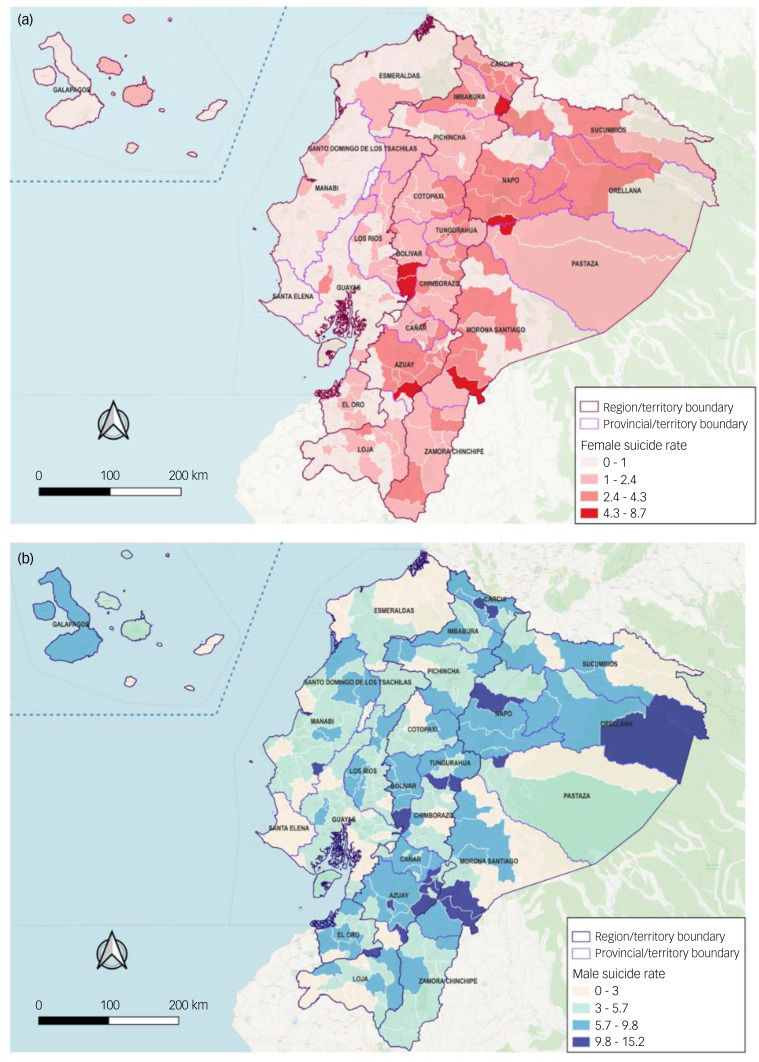
Geographical distribution of adjusted suicide rates (×/100 000) among Ecuador cantons, from 2011 to 2021. (a) Suicide rates in women. (b) Suicide rates in men.

Conversely, some provinces and cantons in Ecuador demonstrated comparatively lower suicide rates. The provinces with the lowest suicide rates were Santa Elena, Esmeraldas and Guayas, with rates of 2.35/100 000, 3.16/100 000 and 3.53/100 000, respectively. At the canton level, Guayaquil, Durán and Quito displayed the lowest suicide rates, with rates of 5.01/100 000, 7.5/100 000 and 7.92/100 000, respectively (Table 1 and Fig. 2).

### Altitude analysis

**Fig. 3:**
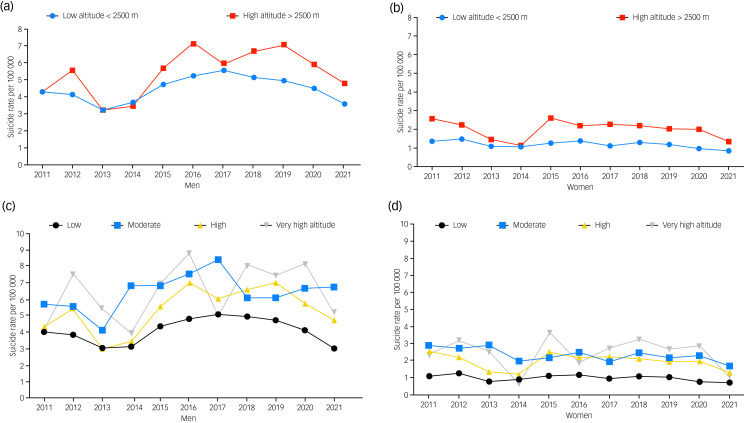
Gender differences in suicide rates in Ecuadorians at different altitudes from 2011 to 2021.

Over the past few years, suicide rates have remained relatively stable among men and women, only decreasing in 2013 and 2014. Furthermore, rates for both genders were lower at low altitudes in all years between 2011 and 2021 (Fig. 3).

When comparing low and high altitude, significantly higher suicide rates were found among people living in rural areas at high altitude than at lower altitudes. With respect to marital status, married, divorced and widowed people at high altitude showed significantly higher suicide rates (*P* < 0.05). In our study, we observed significant differences in suicide rates among individuals living at low (<2500 m) and high altitudes (>2500 m), particularly among certain ethnic groups and levels of education. Specifically, among Indigenous, Mestizo and Montubios ethnic groups, suicide rates were significantly higher at higher altitudes. For Indigenous individuals, for instance, suicide rates were 121% higher at higher altitudes, whereas for Mestizo and Montubios groups, the rates were 35 and 53% higher, respectively. As for education, we noticed a similar trend. Individuals with a higher (technical and higher university) level of education exhibited significantly higher suicide rates at high altitudes, with an increase of 129 and 126%, respectively. This trend was also seen among individuals who had completed secondary and basic education. Moreover, the suicide rates were significantly higher among both men and women residing at high altitudes, with an increase of 22 and 70%, respectively, compared with those living at low altitudes. The same was true for individuals living in rural areas, where the suicide rate was 119% higher at high altitudes (Table 2).

**Table 2:** Differences in suicide rates by rates per 100 000 inhabitants for low and high altitude

	Low altitude (<2500 m)		High altitude (>2500 m)				
	*n*	Rate	*n*	Rate	Difference	s.e. of difference	*P-*value
Gender							
Men	5689	4.5	2989	5.4	22%	0.47	0.0502
Women	1502	1.2	1096	2.0	70%	0.16	**<0.001**
No information	2	Not applicable	2	Not applicable	Not applicable		
Residence							
Rural	1328	1.0	1253	2.3	119%	0.33	**0.0011**
Urban	5095	4.0	2375	4.4	9%	0.70	0.6276
No Information	770	Not applicable	455	Not applicable	Not applicable		
Civil status							
Married	1429	1.1	1104	2.0	80%	0.22	**<0.001**
Divorced	251	0.2	184	0.3	70%	0.06	0.0265
Single	4025	3.2	2232	4.1	29%	0.54	0.1021
Common law	1137	0.9	359	0.6	−28%	0.40	0.5369
Widower/widow	160	0.1	107	0.2	55%	0.03	**0.0366**
No information	189	Not applicable	99	Not applicable		Not applicable	
Ethnicity							
Afro Ecuadorian	379	0.30	91	0.19	−39%	0.18	0.4569
Indigenous	442	0.35	420	0.77	121%	0.07	**<0.001**
Mestizo	5878	4.58	3415	6.20	35%	0.61	0.0152
White	56	0.21	24	0.21	0%	0.01	0.9445
Montubios	153	0.04	14	0.06	53%	0.02	**<0.001**
Other	15	0.11	5	0.03	−71%	0.00	0.5165
No information	268	0.01	116	0.020	37%	0.07	0.9998
Education level							
Illiterate	331	0.26	135	0.27	4%	0.06	0.8222
Literacy centre	58	0.04	31	0.06	37%	0.01	0.4133
Primary education	2195	1.75	1061	1.96	12%	0.33	0.5280
Secondary education	1447	1.14	909	1.68	47%	0.28	0.0654
Basic education	1492	1.13	886	1.57	39%	0.45	0.3426
High school education	845	0.64	507	0.98	54%	0.30	0.4021
Baccalaureate cycle	39	0.04	28	0.07	66%	0.02	0.2119
Technical education	226	0.24	222	0.56	129%	0.10	0.0297
Postgraduate	1	0.00	13	0.05	513%	0.07	0.1202
Non-university education	29	0.05	25	0.15	168%	0.01	0.4541
Higher university	93	0.25	90	0.57	126%	0.03	0.3375
No information	435	0.34	178	0.32	−7%	0.09	0.7415

In this analysis, the Bonferroni correction was applied because 26 valid comparisons were identified. As a result, the significance level has been adjusted to approximately 0.0019.

Statistically significant *P*-values are shown in bold.

According to the ISMM classification, the overall mean suicide rate for elevations below 1500 m was found to be lower compared with moderate, high and very-high altitudes (*P* < 0.001) In the comparison of suicide rates by gender between low altitude and other altitudes, the rates were significantly lower for individuals living at low altitudes (*P* < 0.05). As for the different age groups, the suicide rates at low altitudes were higher for school-aged, adolescent, young adult and elderly individuals compared with those at other altitudes. Furthermore, there were significant differences between the suicide rates of school-aged, adolescent and young adult groups living at low altitudes (<1500 m) compared with those living at moderate altitudes (1500 m to 2500 m), as illustrated in Supplementary File 2.

On the other hand, the analysis revealed differences in suicide rates when comparing low altitude (<1500 m) with higher altitudes (moderate, high and very high) among residents of rural areas (*P* < 0.001). Marital status also appears to have an inverse relationship with suicide rates, with the mean rate of cases at low altitude being higher for all marital status groups compared with other altitudes. However, statistically significant differences were found among married, single and widowed individuals (*P* < 0.05). The ethnic group most affected by suicide was the Mestizos, with a mean suicide rate at low altitude (<1500 m) that was approximately four times higher than at higher altitudes (*P* < 0.05). Further details can be found in Table 3.

**Table 3:** Differences in mean suicide rates by population characteristics, according to the International Society of Mountain Medicine classification

	Reference group		Comparative group								
	Low altitude (<1500 m)		Moderate altitude (1500–2500 m)			High altitude (2500–3500 m)			Very-high altitude (3500–5500 m)		
	*n*	Rate	*n*	Rate	*P-*value	*n*	Rate	*P-*value	*n*	Rate	*P-*value
Overall	5529	2.55	1662	4.38	**<0**.**001**	3731	3.67	**<0**.**001**	354	4.41	**<0**.**001**
Gender											
Men	4468	4.11	1221	6.42	**<0**.**001**	2732	5.36	0.016	257	6.40	**<0**.**001**
Women	1061	0.98	441	2.34	**<0**.**001**	999	1.98	**<0**.**001**	97	2.43	**<0**.**001**
Residence											
Rural	758	0.70	570	3.01	**<0**.**001**	1102	2.16	**<0**.**001**	151	4.13	**<0**.**001**
Urban	4156	3.84	939	5.47	0.111	2213	4.81	0.453	162	4.07	0.768
No information	615	1.51	153	4.31		416	2.92		41	10.99	
Civil status											
Married	1029	9.57	400	2.13	**<0**.**001**	993	1.96	**<0**.**001**	111	2.78	**<0**.**001**
Divorced	185	1.24	66	0.35	0.003	171	0.33	0.007	13	0.59	0.377
Single	3134	16.82	891	4.70	0.006	2051	4.04	0.042	181	4.51	0.017
Common law	923	3.87	214	1.10	0.966	332	0.64	0.583	27	1.04	0.629
Widower/widow	111	1.32	49	0.26	0.005	92	0.18	0.015	15	0.46	0.009
No information	147	0.71	42	0.22	0.011	92	0.18	0.179	7	0.51	0.655
Ethnicity											
Afro Ecuadorian	329	0.71	50	0.30	0.83	86	0.19	0.467	5	0.48	0.333
Indigenous	304	3.01	138	0.74	0.001	385	0.77	**<0**.**001**	35	0.87	0.001
Mestizo	4463	28.46	1415	7.43	**<0**.**001**	3111	6.09	0.004	304	7.57	**<0**.**001**
White	45	0.29	11	0.07	0.189	21	0.06	0.935	3	0.82	0.659
Montubios	146	0.29	7	0.07	**<0**.**001**	12	0.03	**<0**.**001**	2	0.27	0.029
Other	13	0.02	2	0.06	0.873	5	0.02	0.622	0	0.00	0.002
No information	229	0.72	39	0.21	0.988	111	0.22	0.917	5	0.35	0.197
Education level											
Illiterate	279	1.32	52	0.31	0.769	122	0.27	0.777	13	0.60	0.598
Literacy centre	49	0.29	9	0.11	0.894	28	0.06	0.457	3	0.28	0.450
Primary education	1706	11.28	489	2.63	0.025	939	1.88	0.397	122	3.07	0.002
Secondary education	1080	8.08	367	1.96	0.009	821	1.64	0.025	88	2.22	0.004
Basic education	1180	5.74	312	1.76	0.253	826	1.59	0.243	60	1.78	0.442
Middle/high school education	620	3.70	225	1.26	0.087	470	0.99	0.248	37	1.41	0.297
Post-Baccalaureate cycle	30	0.37	9	0.11	0.31	25	0.08	0.211	3	0.28	0.247
Higher education	146	1.39	80	0.59	0.017	211	0.58	0.008	11	0.77	0.286
Postgraduate	1	0.01	0	0.00	0.329	13	0.06	0.121	0	0.00	0.329
Higher non-university education	20	0.24	9	0.16	0.365	24	0.16	0.347	1	0.26	0.806
Higher university	61	1.72	32	0.58	0.254	84	0.57	0.242	6	0.79	0.407
No information	357	1.13	78	0.42	0.291	168	0.33	0.954	10	0.46	0.424

The Bonferroni correction has been applied to adjust for multiple comparisons in this study, considering 78 comparisons across various altitude categories and demographics. This correction changes the significance level to 0.00064, accounting for the increased risk of type I errors in multiple tests.

Statistically significant *P*-values are shown in bold.

#### Suicide methods

The most used suicide methods, according to the altitude analysis, were hanging and the use of pesticides (Supplementary File 3). Regarding cases of suicide by hanging, high-altitude cases showed a higher rate (54.6/100 000), whereas the rate of suicide by pesticides was higher at low altitudes (5.6/100 000). As for the analysis according to the ISMM classification, similarly, the most recorded methods of suicide were hanging and pesticides. However, according to the ISMM classification, the highest suicide rate for hanging was seen at moderate altitude (57.6/100 000) and very-high altitude (70.2/100 000). On the other hand, the highest suicide rates for pesticides were seen at low altitude (5.7/100 000) and moderate altitude (5.3/100 000) (see Supplementary File 3).

### Burden of suicide

Regarding YLL from suicide in Ecuador from 2011 to 2021, the analysis by altitude showed that the cases registered at altitudes below 2500 m had the highest YLL, and within this group, men were the most affected, with 157 600 YLL. However, the increase in YLL per 100 000 inhabitants was higher for women (71.6%) than for men (29.30%) when comparing the rates of those living at low altitude and those living at high altitude. When considering the ISSM classification, the highest YLL occurs at low altitude, and YLL is greater for men than for women at any altitude. When considering YLL per 100 000 inhabitants, the number is significantly lower at low altitude when compared with all the other altitude levels (moderate, high and very high). Additionally, when evaluating the method of suicide, we observed that the method that resulted in the most YLL in both men and women was hanging (213 598.24 YLL), followed by pesticides (25 565.29 YLL). Furthermore, in cases of alcohol, drowning and sharp objects, significantly higher numbers of YLL were observed in YLL for men compared with women (Table 4).

**Table 4:** Years of life lost caused by suicide in Ecuador according to altitude and cause of suicide, stratified by gender

	Men	YLL/ 100 000	Women	YLL/ 100 000
Altitude (low versus high)				
<2500 m	157 600	2663	42 130	711
>2500 m	82 264	3444	31 036	1220
Altitude (ISMM)				
Low	25 182	497	19 974	398
Moderate	4548	536	3546	391
High	20 453	925	19 246	818
Very high	1356	760	1394	732
Suicide methods				
Alcohol	1325	405	30	21
Blunt objects	150	39	30	4
Crashing cars	30	5	Not applicable	Not applicable
Drowning	1024	325	272	84
Explosives	150	36	60	14
Fire	150	99	151	57
Firearms	14 338	4466	727	152
Gases	542	160	212	28
Hanging	169 619	43 532	43 979	9387
Jumping from heights	3373	1059	1273	428
Jumping from moving cars	60	53	60	29
Medication	2018	476	1636	307
Other	26 417	6526	16 094	2663
Pesticides	17 199	4320	8365	1432
Poison	180	47	Not applicable	Not applicable
Sharp objects	3283	1193	272	137

YLL, years of life lost; ISSM, International society of Mountain Medicine.

## Discussion

This research aimed to analyse the trend and distribution of suicide in Ecuador, and the potential influence of geographic elevation and other demographic factors, such as age, gender and place of residence, on suicide. A total of 11 280 suicide deaths were recorded from 2011 to 2021, with overall annual incidence rates ranging from 6 to 11 per 100 000 people among men and 2–3 per 100 000 among women. These figures, although considerable, are lower than those recorded in other countries in the Americas during the 2015–2019 period, such as Uruguay (18.8/100 000), the USA (14.5/100 000) and Haiti (11.2/100 000).^28^ According to studies conducted in Ecuador over the past decade, the suicide rate, particularly among adolescents, has significantly increased from 11/100 000 to 27/100 000.^3^ Additionally, it is important to note that in a previous report we examined, it was found that from 2001 to 2015, Ecuador officially recorded a total of 13 500 deaths from suicide, with an average of 890 deaths per year, ranking second in terms of suicide rates among Andean nations such as Colombia (6.1/100 000 inhabitants), Peru (5.8/100 000 inhabitants) and Bolivia (18.7/100 000 inhabitants).^3^ Previous reports and the current data also reveal that men are three times more likely to attempt suicide than women, and that men tend to use more lethal and effective methods of suicide than women.^3^

Overall, suicide rates have increased since 2011, primarily driven by a bigger incidence rate among men. Feature often seen in other South American countries.^29^ Likewise, in terms of proportion, men accounted for 76.8% of recorded suicides, similar to those reported during the period 2015–2019 in Colombia, Paraguay, Brazil and countries of Central and North America.^28^ Possible explanations have been proposed for the significant differences among countries in terms of suicide linked to gender, such as cultural acceptance of suicide and psychosocial differences between men and women, including the intentionality of suicidal behaviour.^30^

Our analysis revealed that the age groups most affected by suicide were young individuals between the ages of 15 and 29 years, as well as individuals over the age of 80 years. A report by the Pan American Health Organization indicated that in 2019, the highest suicide rates were seen in the oldest age groups (over 85 and between 80 and 84 years) in the Americas region.^28^ However, it is important to note that the relationship between age and suicidal behaviour is not fully understood, as factors such as attitude toward suicide and help-seeking behaviour can vary among different age groups and may influence suicide rates.^31^ Further research is needed in this area.

In relation to the specific age-related patterns observed, our study identified an unusual peak in suicide rates at age 94 years, with a rate of 40 per 100 000. This peak, although based on a limited number of cases, might be influenced by factors such as the COVID-19 pandemic's impact on the elderly, particularly from 2020 to 2021, and gender disparities in social support networks. Elderly men, who may be more isolated, could be at a higher risk of suicide. This finding, although intriguing, should be interpreted with caution, because of the small sample size and the complex interplay of factors at this advanced age. Further focused research is necessary to understand the specific causes and implications of this observation.

The most used method of suicide between 2011 and 2021 was self-inflicted injury by hanging, which was shown to be the main method of suicide among Ecuadorians, followed by pesticides. The pooled analysis in the period from 2000 to 2017 of the Americas exposed that across the region, the most reported method of suicide was self-inflicted poisoning.^28^ These discrepancies have been observed in other isolated analysis reports. In Peru, Roman-Lazarte et al found that, unlike in previous periods (2004–2013), suicides by hanging increased between 2017 and 2019,^29^ and Bustamante et al reported that hanging was the most used suicide method between 2001 and 2010 in Chile.^32^

When comparing the suicide methods observed among individuals residing at different altitudes, there appears to be a potential accessibility factor at play. This is supported by a study conducted by Lin et al, which found an association between residing in high-altitude areas and an increased likelihood of suicide by jumping, likely because of the relative ease of accessing high-altitude locations.^33^

In our study, we hypothesise that in low-altitude rural areas, particularly in coastal regions of Ecuador, the abundance of food resulting from favourable climatic conditions for agriculture might contribute to lower suicide rates. These areas, with their large plantations and consistent crop yields, may offer a more stable and supportive environment, potentially positively affecting the mental well-being of residents.

Conversely, in high-altitude rural areas, the harsher climate and limited agricultural potential might lead to more challenging living conditions. We speculate that factors such as limited food production, poor housing and the overall harsher environment could influence the mood of the residents.^34^ It's important to note that in Ecuador, the distinction between urban and rural areas can be complex, as many individuals inhabit both environments.

In our study, we noticed that individuals residing in low-altitude geographical areas were more prone to suicide involving the use of pesticides. This could be attributed to the fact that provinces with significant agricultural activities, such as Manabí, Guayas and Los Ríos, are located at lower altitudes, resulting in greater access to pesticides. Additionally, the use of firearms for suicide was more prevalent in low-altitude areas compared with high-altitude areas, potentially because of easier access to firearms in those regions. One article highlights that informal markets in Guayaquil offer easy access to large-calibre weapons and ammunition.^35,36^ These findings suggest that the differences in suicide methods observed across altitudes may be influenced by the availability and accessibility of specific means, emphasising the need for targeted prevention strategies in different geographical areas.

In terms of elevation, the highest number of suicide deaths (absolute number) was observed in Ecuadorian populations residing below 2500 m, a difference that was not observed in the analysis of adjusted suicide rates. When adjusting for population at risk, using the International Suicide Mortality Classification, the highest suicide rates were observed at moderate and high altitudes. Furthermore, when analysing different age groups, statistically significant higher suicide rates were found among school children, adolescents and young adults. This is consistent with previous research by Betz et al^14^ which suggested a relationship between high altitude and suicide risk, taking into account factors such as altitude/hypoxia, demographic situations, mental health, race, Hispanic ethnicity, intoxication, firearm use, and interpersonal or financial problems.^14^

According to a study conducted by Kim et al,^37^ which analysed data from 2005 to 2008, there is a significant positive correlation between suicide rates and altitude in both South Korean and American populations.^38^ Similarly, Kious et al found that relocating to high altitudes (≥900 m) is associated with an increased risk of depression, anxiety and suicidal ideation.^18^ One of the most widely studied theories on the relationship between high altitude and suicide is the effect of hypoxia. It is believed that prolonged hypoxia worsens mood disorders, particularly in individuals who are emotionally unstable.^39,40^ The other component of this hypothesis considers the fact that hypoxia might reduce serotonin production, which could promote anxiety and depression.^41^ In general terms, tryptophan hydroxylase needs molecular oxygen to convert tryptophan to 5-HTP, therefore serotonin production could be hampered at high-altitude locations.^42^ Furthermore, hypoxia has been found to decrease serotonin production in animal models, which is frequently linked to depressive-like behaviour.^43^

Beyond the biological implications of altitude, such as hypoxia, it is crucial to consider other influencing factors. These can encompass socioeconomic issues, isolation, access to mental healthcare, cultural norms and stigma associated with mental health.^44^ For instance, living at higher altitudes might entail less access to mental health services or increased social isolation, both of which can contribute to higher suicide rates. Moreover, cultural aspects and stigma related to mental health vary significantly across different regions and communities, potentially affecting suicide rates. It is worth noting that our study was not designed to thoroughly investigate these elements, but we acknowledge their potential impact on the observed outcomes.

In the context of the global COVID-19 pandemic, it is important to acknowledge its potential impact on mental health and suicide rates. However, a recent study conducted in Ecuador reported that there was no evidence that suicide rates were higher than expected during the first 16 months of the pandemic.^45^ This finding suggests that the pandemic might not have significantly affected our results. Nevertheless, the unique stressors introduced by the pandemic, such as lockdown measures, job loss, social isolation and the associated psychological impact, could still have influenced individual cases of suicide, and thus remain important considerations in this context.

We acknowledge that it is difficult to find a causal association between altitude and suicide; however, we believe that factors that are more common at high altitudes, such as mood swings, the incidence of depression and social isolation, may be linked to the higher number of suicides at altitude. For instance, mood instability and pessimism are often features of patients with suicidal tendencies. High-altitude dwellers from Ecuador seem to be more pessimistic than low-altitude dwellers, and although the prevalence of depression was not studied, mental and health scores are poorer at higher altitudes.^20^

It is interesting to note that there are significant differences in terms of the mechanism used for suicide. Hanging remains the primary mechanism in any region, province and canton, regardless of their elevation; nevertheless, it is evident that firearms are used less frequently in the highlands than at sea level, whereas populations at high altitudes jump from heights more often.

We recognise the limitations from this type of analysis, but we also believe the results of this study will provide additional data on the possible effects of altitude on suicide risk. Finally, the peculiar geography of the Ecuadorian Andes allows this type of analysis, which includes a higher range of elevation than any of the other available reports.

### Limitations

First, it is important to acknowledge that data on suicide attempts were not available for analysis, which could have provided valuable contextual information to better understand the factors contributing to suicide. The lack of such data limits our ability to comprehensively explore the complete spectrum of suicidal behaviour. Regarding the use of publicly available data from INEC databases, it is crucial to recognise that this approach introduces inherent limitations. The quality of the data is contingent on the accuracy and completeness of the initial input, over which our research team had no control. As a result, the possibility of biases arising from data entry errors or incomplete reporting should be considered. Another significant limitation arises from the geographical unit of analysis, the canton, which may have introduced a level of aggregation bias in our findings. The canton-level analysis may obscure variations within cantons, potentially affecting the conclusions drawn about the association between altitude and suicide rates.

Furthermore, our study did not employ multivariate analyses or meta-regression methods, which could have allowed us to control for various canton characteristics, such as mean altitude, gender ratio, mean age, mean income level, mean educational level and population density. By not incorporating these additional factors, our analysis might be limited in its ability to account for potential confounders that could influence the observed relationship between altitude and suicide rates. Despite these limitations, we have taken a careful and rigorous approach to analyse the available data. However, it is essential to recognise that the data's inherent limitations and the methodological choices made may influence the interpretation of our results.

In summary, although our study provides valuable insights into the association between altitude and suicide rates in Ecuador, we acknowledge the limitations discussed above. Future research should aim to address these limitations by incorporating data on suicide attempts, employing more refined statistical methods to control for potential confounders and utilising data sources with greater accuracy and completeness. By acknowledging these limitations, we hope to encourage further research and collaborative efforts to gain a deeper understanding of suicide patterns and risk factors in Ecuador, ultimately contributing to the development of more effective suicide prevention strategies.

In conclusion, this study represents a pioneering effort in investigating the characteristics of individuals who died by suicide in Ecuador between 2011 and 2021. The findings presented here contribute valuable information to the existing literature, particularly in understanding the potential association between altitude and suicide rates in a country with diverse geographical features, including regions above 3500 m. Our results strongly indicate that suicide rates are notably higher in high-altitude areas compared with lower altitudes, with the most elevated suicide rate of 4.1 found at very-high altitudes (>3500 m). Additionally, the predominant method used for suicide across both genders was hanging.

The burden of disease, as measured by YLL per 100 000 inhabitants, is substantially greater in high-altitude regions compared with lower-altitude areas. These observations underscore the significance of altitude as a relevant variable to consider in understanding suicide patterns and risk factors in Ecuador.

As this study is the first of its kind in Ecuador, the findings presented here highlight the necessity for further research to comprehensively explore the multifaceted factors, both behavioural and risk-related, that may contribute to the elevated suicide rates in high-altitude areas. Gaining a deeper understanding of these complex dynamics is essential for designing effective prevention and intervention strategies that can address the unique challenges faced by individuals living in high-altitude regions.

Overall, this study adds to the growing body of knowledge on suicide patterns, and emphasises the importance of continued research efforts to identify and address the underlying factors that contribute to higher suicide rates in high-altitude regions. By promoting further investigations and collaboration among researchers and stakeholders, we can work toward developing evidence-based strategies that aim to reduce suicide rates and promote mental well-being in Ecuador.

## Supporting information

Ortiz-Prado et al. supplementary material Ortiz-Prado et al. supplementary material

## Data Availability

In our study, we did not employ any unique analytic coding that generated the results. We used standard statistical analysis procedures with widely used statistical software. Consequently, we did not create any specific analytic code for this study that can be deposited in a repository. We have made note of this in the manuscript. Data on suicide at different elevations from Ecuador (2011–2021) can be found in the following repository: https://github.com/covid19ec/SuicideData.
