# Supplementary material for: The burden of suicide across different altitudes: 11-year geodemographic analysis conducted in 221 cantons in Ecuador ranging from 0 to 4300 m of elevation
Source: BJPsych Open. 2024 Sep 24;10(5):e161. doi: 10.1192/bjo.2024.736 (PMC11457200; doi:10.1192/bjo.2024.736)
Supplement: Ortiz-Prado et al. supplementary material [file S2056472424007361sup001.docx]

Supplementary Table 1 ICD-10 suicide or intentional self-harm classification according to the National Institute of Statistics and census.

| **ICD-10 Code** | **Means for suicide** | **Categorization of suicide mechanisms by etiologic group** |
| --- | --- | --- |
| **X60** | Intentional self-poisoning by and exposure to nonopioid analgesics, antipyretics and antirheumatics | Suicide by drugs, medicines, and chemicals |
| **X61** | Intentional self-poisoning by and exposure to antiepileptic, sedative-hypnotic, antiparkinsonism and psychotropic drugs, not elsewhere classified |  |
| **X62** | Intentional self-poisoning by and exposure to narcotics and psychodysleptics [hallucinogens], not elsewhere classified |  |
| **X63** | Intentional self-poisoning by and exposure to other drugs acting on the autonomic nervous system |  |
| **X64** | Intentional self-poisoning by and exposure to other and unspecified drugs, medicaments, and biological substances |  |
| **X65** | Intentional self-poisoning by and exposure to alcohol | Suicide by and exposure to alcohol |
| **X66** | Intentional self-poisoning by and exposure to organic solvents and halogenated hydrocarbons and their vapors | Suicide by and exposure to organic solvents and their vapors |
| **X67** | Intentional self-poisoning by and exposure to other gases and vapors |  |
| **X68** | Intentional self-poisoning by and exposure to pesticides | Suicide by and exposure to pesticides |
| **X69** | Intentional self-poisoning by and exposure to other and unspecified chemicals and noxious substances | Suicide by unspecified chemicals and noxious substances |
| **X70** | Intentional self-harm by hanging strangulation and suffocation | Suicide by hanging strangulation and suffocation |
| **X71** | Intentional self-harm by drowning and submersion | Suicide by drowning and submersion |
| **X72** | Intentional self-harm by handgun discharge | Suicide by fire gun |
| **X73** | Intentional self-harm by rifle, shotgun, and larger firearm discharge |  |
| **X74** | Intentional self-harm by other and unspecified firearm discharge |  |
| **X75** | Intentional self-harm by explosive material | Suicide by explosive material, fire, and hot objects |
| **X76** | Intentional self-harm by smoke, fire, and flames |  |
| **X77** | Intentional self-harm by steam, hot vapors and hot objects |  |
| **X78** | Intentional self-harm by sharp object | Suicide by sharp object |
| **X79** | Intentional self-harm by blunt object | Suicide by blunt object |
| **X80** | Intentional self-harm by jumping from a high place | Suicide by jumping from a high place |
| **X81** | Intentional self-harm by jumping or lying before moving object | Suicide by jumping or lying before moving object |
| **X82** | Intentional self-harm by crashing of motor vehicle | Suicide by crashing of motor vehicle |
| **X83** | Intentional self-harm by other specified means | Suicide by other specified and unspecified means |
| **X84** | Intentional self-harm by unspecified means |  |

**
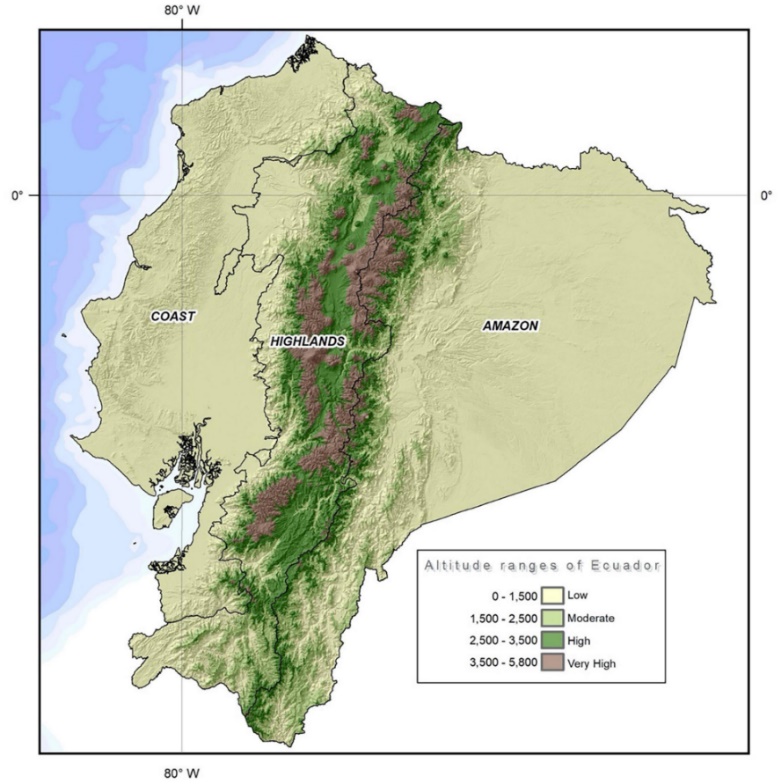
**

Supplementary file 1 Geographic distribution of the equator according to altitude


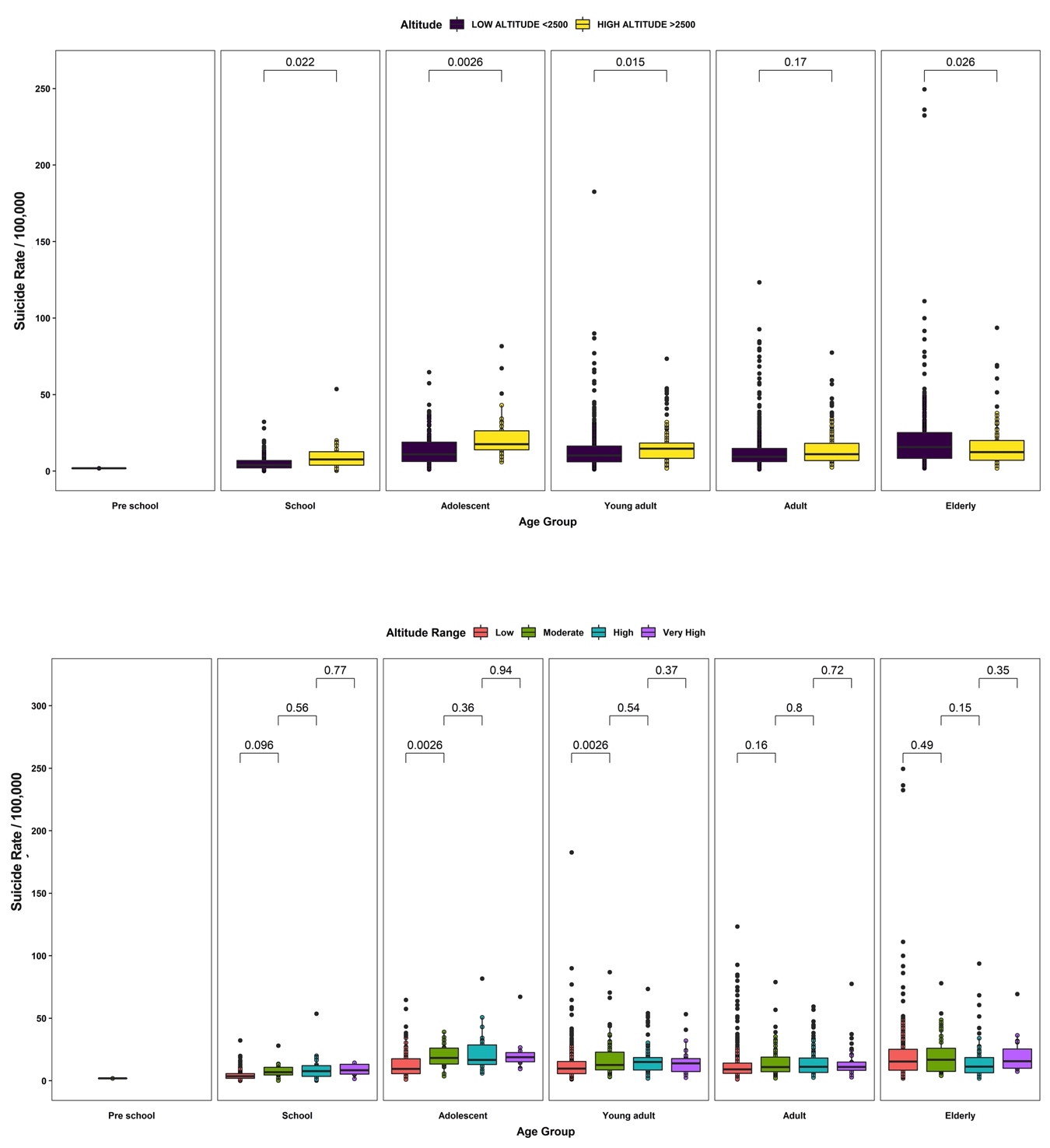


Supplementary file 2 Age groups suicide rate at different altitude. A: Suicide rate at low (<2,500 m) and High (>2,500 m). B: Suicide rate at four-high altitude classification, at low (<1,500m), Moderate altitude (1,500 m to 2,500 m), high altitude (2,500 m to 3,500 m) and very high altitude (3,500 to 5,800 m)


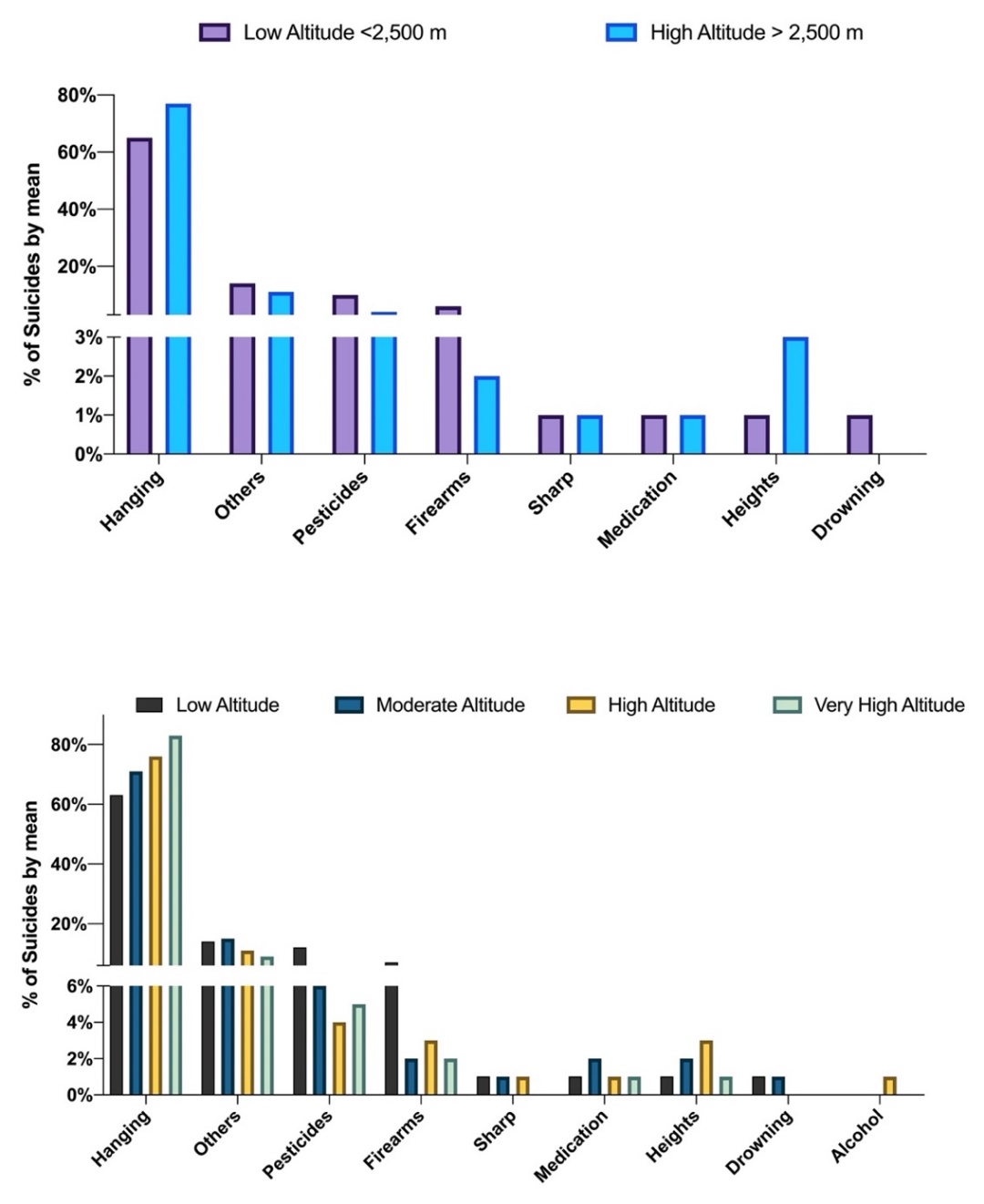


Supplementary file 3 Distribution of suicide rates according to suicide methods
